# Supplementary material for: Molecular subtyping improves prognostication of Stage 2 colorectal cancer
Source: BMC Cancer. 2019 Nov 27;19:1155. doi: 10.1186/s12885-019-6327-4 (PMC6882162; doi:10.1186/s12885-019-6327-4)
Supplement: Supplementary file 1 — Additional file 1: Table S1. Patient demographics and clinical characteristics. Table summarizing clinical and patient characteristics of the cohort analysed in the study. Table S2. Comparison of proportions of Consensus Molecular Subtypes (CMS) and associated gene signatures of colorectal cancers (CRC) from Colorectal Cancer Subtyping Consortium8 and the current New Zealand CRC Predict study. Table summarizing the percentage of the study cohort assigned to each CMS, and the associated gene signatures, compared to the original CMS publication. Figure S1. Overview of enriched biological terms per subtype for differentially up-regulated genes. Illustration of the main molecular signatures associated with each CMS in our cohort [file 12885_2019_6327_MOESM1_ESM.docx]

**Supplementary Table S1.** Patient demographics and clinical characteristics

| **Gender** |  |  |  |
| --- | --- | --- | --- |
| Female |  | 163 |  |
|  | Male |  | 145 |
| **Age** |  |  |  |
|  | <55 |  | 27 |
|  | 56-79 |  | 192 |
| >80 |  | 91 |  |
| **Ethnicity** |  |  |  |
|  | Maori |  | 9 |
|  | Asian |  | 3 |
| European |  | 296 |  |
| **CMS** |  |  |  |
|  | 1 |  | 61 |
|  | 2 |  | 139 |
|  | 3 |  | 39 |
|  | 4 |  | 17 |
| UC |  | 52 |  |
| **Site** |  |  |  |
|  | Rectum |  | 55 |
|  | Left |  | 118 |
|  | Right |  | 135 |
| **Post-op staging** | |  |  |
|  | 1 |  | 53 |
|  | 2 |  | 128 |
|  | 3 |  | 105 |
|  | 4 |  | 22 |
| **Recurrence and metastases** | |  |  |
|  | Positive LN |  | 123 |
|  | Local recurrence | | 14 |
|  | Synchronous Mets | | 18* |
|  | Subsequent Mets | | 56** |
| **Post-operative therapy** | |  |  |
|  | Adjuvant |  | 75 |
|  | Adj + palliative | | 13 |
| Palliative |  | 19 |  |
| **Histology** |  |  |  |
|  | Differentiation | |  |
|  |  | Poor | 53 |
|  |  | Moderate | 171 |
|  |  | Well | 12 |
|  |  | Unknown | 72 |
|  | Mucinous |  | 32 |
|  | LVI |  | 101 |
| EMI |  | 46 |  |
|  | Perineural invasion |  | 37 |

CMS, consensus molecular subtyping; UC, unclassified; LN, lymph node; Mets, metastases; adj, adjuvant; LVI, lymphovascular invasion; EMI, extra-mural invasion

**Supplementary Table S2.** Comparison of proportions of Consensus Molecular Subtypes (CMS) and associated gene signatures of colorectal cancers (CRC) from Colorectal Cancer Subtyping Consortium^8^ and the current New Zealand CRC Predict study.

|  | **CMS1**  **MSI immune** | **CMS2**  **Canonical** | **CMS3**  **Metabolic** | **CMS4**  **Mesenchymal** | **Unclassified** |
| --- | --- | --- | --- | --- | --- |
| **CMS study^8^** | 14% | 37% | 13% | 23% | 13% |
|  | Immune  infiltration and activation | Wnt and Myc activation | Metabolic  deregulation | Stromal infiltration,  TGFβ activation,  angiogenesis |  |
| **NZ CRC Predict study** | 19% | 47% | 12% | 6% | 16% |
|  | Immune  infiltration and activation | Neural activation | Metabolic  deregulation | Stromal infiltration,  TGFβ activation,  angiogenesis |  |


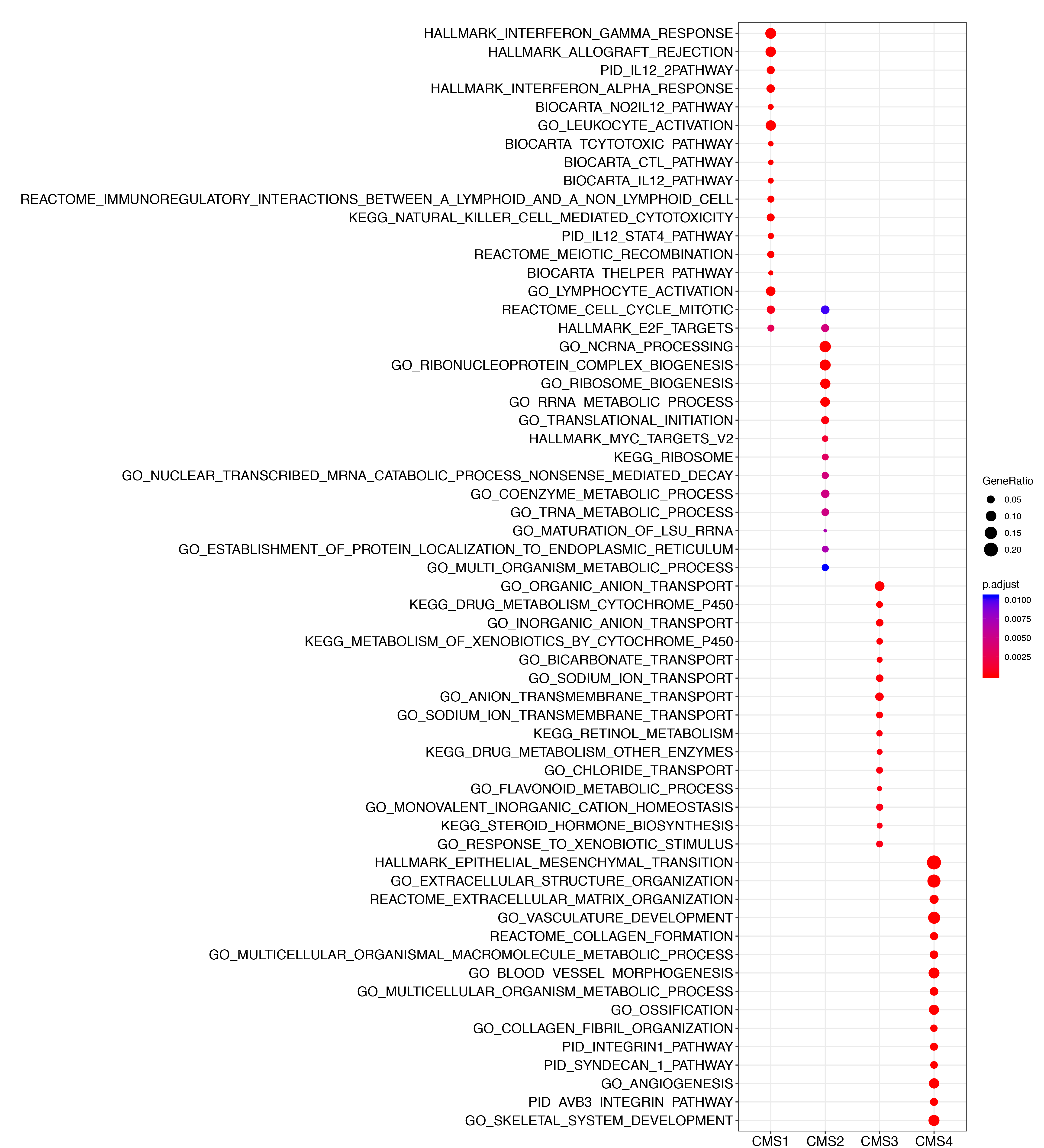


**Supplementary Figure S1**. Overview of enriched biological terms per subtype for differentially up-regulated genes.
